# A Neurosociological Theory of Culturally and Structurally Situated Cognition and Ethno-Racial Stress

**DOI:** 10.3389/fsoc.2021.695042

**Published:** 2021-06-11

**Authors:** Rengin B. Firat

**Affiliations:** Department of Sociology, University of California, Riverside, CA, United States

**Keywords:** neurosociology, ethno-racial stress, values, culture, coping, fMRI, agentic, communal

## Abstract

A longstanding body of literature reveals that experiences of discrimination and exclusion lead to health disadvantages by increasing physiological stress responses both in the body and the brain. However, a sociological view that takes into account structurally and culturally shaped biological processes is missing from the literature. Building on recent literature from the sociology of morality and values and the dual process model of culture, this paper proposes and provides preliminary evidence for an applied theory of culturally situated moral cognition as a coping mechanism with ethno-racial stress. I focus on values as they help cope with ethnicity and race related stress such as discrimination. Using functional neuroimaging data, I offer evidence that values operate through both explicit (controlled and conscious) processes recruiting brain regions like the dorsal prefrontal cortex, and implicit (automatic and non-conscious) processes recruiting regions like the ventromedial prefrontal cortex, to help cope with exclusion and discrimination.

## Introduction

A longstanding body of literature reveals that experiences of discrimination lead to health disadvantages by increasing physiological stress responses such as blood pressure, cardiovascular reactivity and heart rate (Brondolo et al., 2003; Pascoe and Smart Richman, 2009). However, not all marginalized people are affected the same way from the negative effects of discrimination and social exclusion (Hughes et al., 2014; Nazroo, 2003). Psychologists have focused on various personality factors like self-efficacy or vigilant coping as protectors from stressors and stigma (Bandura, 1998; McAuley, 1992); yet a sociological view that takes into account structurally and culturally shaped biological processes is missing from the literature. This paper seeks to fill this gap by proposing a new, neurosociological theory of structurally and culturally situated moral cognition (Firat and McPherson, 2010; Firat and Hitlin, 2012) and the ways this moral cognition moderates the relationship between ethno-racial stressors and stress responses in the body and the brain.

This paper argues that cognitive sociology and the newer research area “neurosociology” have the potential to add the literature on health disparities by linking micro and macro foundations and detailing the ways our minds and bodies reproduce social structure and inequalities (Cerulo, 2002; Cerulo, 2014; Franks, 2010; Ignatow, 2007; Lizardo, 2014; TenHouten, 1997). This neurosociological approach distinguishes itself from a “sociology of neuroscience” framework, which takes more of the role of a critique of neuroscientific empirical work alongside other developments in science and technology (von Scheve, 2011). While the critiques and examinations of the knowledge and practices in neurosciences form a new, growing body of scholarship called “critical neuroscience” and are abundant (e.g., Bruder, 2019; Choudhury et al., 2009; Schleim, 2014; Slaby, 2010), a complementary neurosociological approach incorporating neurological and biological processes into sociological theorizing and empirical work is largely missing from the literature (von Scheve, 2011). The rudimentary connections between morality and human evolution and neuroanatomy were broadly made elsewhere (Turner, 2014). This paper attempts to contribute to these efforts towards an integrated, transdisciplinary science of morality (Firat and McPherson, 2010; Firat and Hiltin, 2012; Jeffries, 2014) by offering a specific neurosociological theory of moral cognition as it relates to inter-ethnic stress.

Building on a growing body of recent literature on moral and cognitive sociology (e.g., Hitlin and Vaisey, 2010, Hitlin and Vaisey, 2013; Vaisey, 2008) and the long standing theoretical tradition of Social Structure and Personality (Pearlin, 1989; Thoits, 2006), in this paper, I argue that morality and values provide “an internal moral compass” (Hitlin and Piliavin, 2004: 362) for coping with discrimination. While previous research has looked at the ways values influence a variety of behaviors and attitudes including political behavior, pro-environmental and prosocial behavior (Barnea and Schwartz, 1998; Schultz and Zelezny, 1998; Schwartz, 2010), much less research has been concerned with behavioral effects of morality and values, especially within the health context.

Drawing from recent literature from the sociology of morality and values and the dual process model of culture and moral cognition (Miles et al., 2019; Vaisey, 2008), I propose and empirically illustrate an applied neurosociological theory of culturally situated moral cognition as a coping mechanism with ethno-racial stress. I focus on values as internalized bits of culture that motivate behavior in line with moral worldviews and help cope with ethnicity related stress such as discrimination (e.g., Firat 2016). This is not to say that culture is a fixed and uniform object shared homogenously by all members of groups; this cultural essentialist view is criticized by many (Grillo, 2003; Modood, 1998; Wikan, 1999). Rather, I view culture and morality as cognition or “schema” through which macro level institutions and structures are manifested (Hitlin, 2014) with different degrees and variations depending on individuals positions in the society as well as personal and inter-personal experiences (Firat and McPherson, 2010). This is more akin to the culture as “patterned practice” view offered by Roepstorff et al. (2010), whereby brain activity generated during experimental tasks can reveal patterns of social interaction.

Furthermore, as put forward by recent theorizing in sociology of morality (Vaisey, 2008; Miles et al., 2019), this paper proposes that these different cultural manifestations and patterned practices operate through both explicit (controlled and conscious) and implicit (automatic and non-conscious) processes. The implicit mechanisms through which values moderate ethnicity related stress are largely unknown due to methodological limitations of traditional survey and interview techniques. This paper addresses this limitation by incorporating neuroscientific functional Magnetic Resonance Imaging methods to capture the subtle mental and bodily dimensions of the relationship between values and ethno-racial stress.

## Ethno-Racial Health Disparities

A large body of literature has focused on the effects of living in ethnically and racially diverse societies. This research suggests that while many civil rights have been extended to ethnic/racial minorities and communities are getting increasingly diverse, ethnic diversity is linked to detrimental individual and community outcomes such as decreased social trust and civic life (Putnam 2007). Furthermore, several studies show that ethnic/racial diversity has important connections with health outcomes, especially for minorities. Members of ethnic and racial minority communities show poorer subjective and objective outcomes of well-being and health. For example, African Americans report lower levels of subjective well-being (Thoits and Hewitt 2001), show increased obesity rates (Flegal et al., 2012), hypertension prevalence (Egan et al., 2010) and even higher mortality rates (Geronimus et al., 1996; Kohcanek et al., 2004) than their White counterparts in the US. There is a higher prevalence of mental health problems (e.g., depression, anxiety) among immigrants and refugees in the United States and other Western nations (Fox et al., 2001; Pumariega et al., 2005; Storch and Poutska 2000).

Disparities in socio-economic status are proposed as a fundamental cause of health inequalities as lack of access to resources is linked to various diseases and higher mortality rates (Phelan, Link, and Tehranifar 2010; Phelan and Link 2013) and thus help explain ethnic/racial health disparities (Braveman et al., 2010; Nazroo and Williams 2006; Nazroo, 2003). However, there are important inter-personal mechanisms like stigma and discrimination that account for an significant portion of the effects of socio-economic status on racial/ethnic health outcomes (Hatzenbuehler, Phelan, and Link 2013; Morales et al., 2002; Mays et al., 2007; Paradies 2006; Williams et al., 1997). Research has shown that experiences of discrimination diminishes physical and mental health (Pascoe and Smart Richman, 2009; Williams and Mohammed 2009; Williams et al., 2003). Racist and discriminatory encounters are correlated with anxiety and depression and substance use (e.g., alcohol and tobacco) among African Americans (Brown et al., 2000; Landrine and Klonoff 1996; Sanders-Phillips et al., 2014).

One mechanism through which discrimination and stigma influence negative health outcomes is by heightening stress responses including blood pressure, heart rate and cardiovascular reactivity during interactions with members of different racial and ethnic group members (Brondolo et al., 2003; Clark, 2000; Harrell et al., 2003; Williams, 2012). Previous research has shown that ethno-racial/racial encounters increase stress and anxiety through cortisol changes and cardiovascular reactivity (Mendes and Koslov, 2013) as well as activation in brain regions related to feeling physical pain, i.e. dorsal anterior cingulate cortex (dACC) (Eisenberger, 2003; Eisenberger and Lieberman, 2004). Cumulative exposure to negative ethno-racial interactions and discrimination over time overload the body’s allostatic load—capacity to react and adjust to challenges and stress through physiological responses such as blood pressure, cortisol activity and cardiac output (McEwen and Stellar 1993; McEwen 1998; 2000; 2004; 2005). Allostatic load and cumulative stress have detrimental impact on health especially in members of minority communities who are exposed to repeated negative life events.

However, despite evident disparities in health, some ethnic minority groups have better health than others. For example, Hispanics in the United States have better health status than Whites despite having lower socio-economic status, lack of access to health care resources and experiences of discrimination (Morales et al., 2002). A White ethnic group, Jewish Americans, rated their health lower than non-Jewish White Americans (Pearson and Geronimus 2011). White Americans report higher rates of depression than African Americans (Breslau et al., 2006; Dunlop et al., 2003; LaVeist et al., 2014). Moreover, minority status predicts eudemonic well-being—having a purpose and meaning in life (Ryff et al., 2003). Similarly, both first and second generation Turkish immigrants in Germany have lower mortality rates than (almost half of) their German counterparts, a phenomenon not entirely explained by neither selection (healthier migrants flowing into the host country) nor attrition (less health migrants leaving the host country) effects (Razum et al., 1998). I suggest that some of these contradictory patterns can be reconciled by looking into neurosociology of cultural cognitive coping styles and values, which provides a more refined framework that takes into account inter-individual as well as inter-group variables of social experiences.

## Values and Their Dual Implicit/Explicit Mechanisms

Sociologists of culture and morality have been increasingly paying attention to values as core components of moral culture binding people and motivating behavior (e.g., Longest et al., 2013; Vaisey and Miles 2014; Wuthnow 2008). At the societal level, values are aggregate world views shaped historically by political, cultural and socio-economic conditions (Inglehart 2015; Inglehart and Welzel 2005). At the individual level, values are internalized versions of these broader worldviews; they are relatively stable over the course of one’s life and serve as abstract, cognitive systems directing behavior towards desirable goals in line with their moral viewpoints (Hitlin 2003; 2008; Howard and Renfrow 2006; Rokeach 1973; Schwartz, 1992; 1994). Values are more abstract than attitudes; they transcend beyond situations and events (Rokeach 1973). Contours of values are formed in childhood (and might shift slightly throughout life) based on various cultural and social structural positions people hold (Kohn 1989; Kohn and Schooler 1982; Longest et al., 2013). Studying values not only provide an understanding of cultural differences between social groups (Lamont 1992; 2000), they also provide insights into inter-personal differences as they influence information processing at the individual level (Dehue, McClintock, and Liebrand 1993; McClintock and Liebrand 1988).

While all humans share certain values to some extent (see Turner 2014 for an evolutionary account of human morality), the rank ordering of the values vary depending on various cultural and structural factors. For example, according to one of the most established and sociologically most adopted (e.g., Hitlin 2003; Vaisey and Miles 2014; Firat 2016; Firat et al., 2018) theories of values, Schwartz values theory (Schwartz, 1992; 1994; Schwartz and Bilsky 1987), there are ten basic, general value orientations that have evolved in response to basic conditions of human existence including coordinated social interaction and survival and well-being of the groups (Schwartz, 1992). These ten value orientations, while measured separately, can ultimately be grouped under four general dimensions: self-transcendence (universalism and benevolence), self-enhancement (power, and achievement), openness to change (stimulation, self-direction and hedonism) and conservation (security, traditionalism and conformity) dimension (Bardi and Schwartz, 2003; Schwartz and Bardi 2001; Schwartz and Boehnke 2004). In line with earlier theorizing and research (Firat et al., 2017), these values can be organized under two moral domains: agentic vs communal. These broader conceptions are also in line with the collectivistic vs individualistic value distinctions in cross-cultural psychological research (Hofstede 2001; Markus and Kitayama 1991; Triandis 2001); yet carry a broader meaning without stigmatizing one or the other dimension.

Behavioral correlates of agentic values (such as self-enhancement or openness) usually hinge on taking charge and direction, influencing others behavior and opinions (Bardi and Schwartz, 2003) but also taking generative, intentional action such as environmentally responsible consumption (Urien and Kilbourne, 2011). Being open and eager to new experiences, ideas and adventures (Bardi and Schwartz, 2003; Schwartz, 1992) guide behavior in the direction of social change such as political activism or individual change such as adapting to new media technologies (Besley, 2008; Firat, 2014). Agentic values contribute to the capacities for responding to the environment in novel ways by bringing one’s attention to previously ignored approaches to problem solving, adopting new behavioral strategies and enhancing effectiveness of existing strategies (Firat et al., 2017). Based on sociological theorizing, the motivation for agency provides people with an “imaginative recomposition and critical judgment’’ to ‘‘reframe their relationships to existing constraints’’ (Emirbayer and Mische 1998:1010; see also Sewell and William 1992). Research suggests that agentic orientations contribute to the flourishing of human health by providing capacities to formulate adaptive strategies during difficult situations and eventually help change habits and choices towards healthier outcomes (Bandura, 1998, Bandura, 2004, Bandura, 2005; Welzel and Inglehart, 2010). Thus, they likely contribute to the flourishing of human health, especially in the face of negative life events, by providing capacities to formulate adaptive strategies and eventually help change habits and choices towards healthier outcomes (Bandura, 1998, Bandura, 2005; Welzel and Inglehart, 2010). I argue that this adaptive reimagination and reframing—or the agentic value orientation-is an explicit, conscious effort because it relies on secondary reflection on the initial imagination or thoughts, thus slower cognition.

Communal values (self-transcendence vs. self-enhancement dimension) revolve around cultivating prosocial concern and interest for the welfare of others and the overall community over self or power related advantage. From an evolutionary point of view, the capacities for altruism, empathy and reciprocity and the associated emotional states provided an evolutionary advantage and added to the adaptive fitness of humans by maneuvering complex social organizations (Turner, 2014). People who are higher on communal value orientation, for example, have higher levels of generalized trust (Vyrost, Kentos, and Fedakova 2007) and more inclined to take environmental and other pro-social action (Karp 1996; Schwartz, 2010). Communal values are aligned with humans need and motivation to build coalitions (Kurzban, Tooby, and Cosmides 2001; Cosmides, Tooby, and Kurzban 2003). This evolved capacity, I argue, operates mostly through implicit mechanisms (while might also be readily available for consciousness) as detecting coalitions (or friends vs rivals) and building a sense of coalitional security and safety (see Boyer, Firat, and van Leeuwen 2015) and potentially rely on automatic categorization and identification of social groups (see also Tajfel 1978, 1982; Tajfel and Turner 1979, 1986; see Abrams and Hogg 2004). Thus, a communal value orientation, and its attitude correspondence in strong sense of coalitions, will act fast through automatic processes to shield people from the stressful effects of inter-group interaction.

The implicit vs. explicit mechanisms of communal or agentic values (or any other values whatsoever) have not been investigated so far, leaving an important gap in identifying the mechanisms for coping with ethno-racial stress, which this paper addresses.

## Values as Coping Mechanisms With Ethno-Racial Stress

This paper proposes that culture provides cognitive buffering resources for ethno-racial stress through two value mechanisms: one attenuating felt social pain via communal values and another providing more active and adaptive strategies to these difficult situations through agentic values (see also Bandura, 1998; Bandura, 2005). The limited literature on the moderating role of values on ethnicity related stress also supports these arguments. Mostly based on the cross-cultural theory of values distinguishing between collectivistic vs individualistic values (e.g., Hofstede 2001; Markus and Kitayama 1991; Triandis 2001), research focusing on communalistic values like collectivism among Asians and familism and respect for elderly among Mexican Americans found that these values dampened the negative effects of exclusion and discrimination (Berkel et al., 2010; Delgado et al., 2011; Iwamoto and Liu 2010; Yeh et al., 2006). Studies found that collectivistic individuals did not respond as intensely to social exclusion, helping recovery from ostracism (Pfundmair et al., 2015; Ren et al., 2013). For example, Mexican American values such as familism and respect for elderly foster a disposition for prosociality that serve as emotional support in response to race-related negative life events like discrimination (Brittian et al., 2013; Knight, Cota, and Bernal 1993; Knight and Carlo 2012). This view also fits with cross-cultural research that identified self-transcendence (and openness) as so-called healthy values that facilitate positive mental health (Cohen and Shamai 2010; Jensen and Bergin 1998; Sortheix and Lonnqvist 2014). Living up to cultural values and norms also reduce stress indicators like arterial blood pressure among the darker skinned people (i.e., Blacks in Southern US and Brazil) (Dressler and Bindon, 2000; Dressler et al., 2005).

The protective role of agentic values against the negative effects of ethno-racial interactions is also supported by some literature that mainly focused on psychological constructs such as self-esteem or vigilant coping. While these constructs are not value orientations, they are aligned with agentic orientation’s core dimensions such as being able to take charge and manipulate one’s surroundings; and, thus offer some insights into how agentic values might help cope with stress. Accordingly, research shows that self-esteem protects people from negative social threats and stress (Rector and Roger 1996; 1997) through a vigilant coping style that seeks new, instrumental information and provides active and problem focused solutions in the face of ethno-racial stress and discrimination (Rector and Roger 1996; Tynes et al., 2012). For example, African American adolescents with higher self-esteem showed lower anxiety when faced with online discrimination than those with lower self-esteem (Tynes et al., 2012). Furthermore, vigilant coping style diminished negative mental health outcomes among Korean immigrants in Toronto (Noh and Kaspar 2003) and African Americans (LaVeist et al., 2014). Attribution theory in psychology also offers some evidence suggesting that attributions about the causality and controllability of stigma and discrimination moderate well-being (Crocker and Major 1989; Schmitt and Branscombe 2002). Research directly focusing on values also supports these findings with cross-cultural survey data by demonstrating that openness to change value orientation enhances well-being (Bobowik et al., 2011; Cohen and Shamai 2010; Georgellis, Tsitsianis, and Yin 2009; Sagiv and Schwartz 2000).

In summary, the literature on the social psychological processes of exclusion and inter-group contact have identified two important, seemingly juxtaposed, cultural cognitive resources: communal vs. agentic values (e.g., Sagiv and Schwartz 1995; 1998). I suggest that these seemingly contradictory results can be reconciled through a dual cultural coping mechanism: 1) an implicit motivational coping attenuating felt social pain via communal values and 2) another explicit coping mechanism providing more active and adaptive strategies to these difficult situations through agentic values (see also Bandura 1998; Bandura, 2005).

## Proposed Theory: Neural Dissociation of the Correlates of Communalistic vs Agentic Values

In the proposed framework here, 1) communal values provide a sense of coherence and community that serve as collective resources promoting positive adjustment and resilience, while 2) agentic values contribute to being able to respond to the environment in novel ways by paying attention to previously ignored approaches to problem solving and enhancing effectiveness of coping. While the effects of these value systems on neurological responses to ethno-racial stress have not been directly researched so far, several other studies that focused on similar moderators or potential behavioral manifestations of these values have found evidence in this direction. For example, cross-ethnic/racial friendships reduced stress hormone reactivity in participants who were implicitly prejudiced or highly concerned of out-group rejection (Page-Gould et al., 2008), predicted faster recovery from stress-related respiratory and hormonal reactivity (Page-Gould et al., 2010), and these positive effects were carried on to interactions with novel other-ethnicity members (Page-Gould et al., 2010). Similarly, several factors such as secure attachment style (DeWall et al., 2011), essentialist beliefs about groups, perceptions of discrimination (Masten, Telzer, and Eisenberger 2011), essentialist beliefs about groups (Bernstein et al., 2010), racial group membership and implicit bias (Krill and Platek 2009) were also found to be moderating neural responses to social exclusion and inter-racial contact. Therefore, I expect communal and agentic values to reduce sympathetic (“fight” or “flight”) stress responses such as respiration, heart rate and skin conductance and increase parasympathetic responses that prepare the body to return to the homeostatic state like increasing heart rate variability through recruiting different brain regions for explicit and implicit coping.

Sympathetic and parasympathetic nervous systems are both components of the autonomic nervous system, which regulates the automatic and mostly non-conscious activity of the body. While sympathetic nervous system responds to stressful or physically arousing situations (aka “fight or flight” response) by increasing skin perspiration, respiration and heart rate, parasympathetic nervous system calms the body down or is involved in regular/ordinary activity such as slowing heart rate and respiration. These responses are down or up regulated (through feedback loops) by the central nervous system activity in the brain.

Relying on a functional dissociation in the human prefrontal cortex (see Firat, 2019; also Lieberman 2007), this paper proposes that the ventromedial prefrontal cortex (vmPFC) and the dorsolateral prefrontal cortex (dlPFC) delineate between the implicit and explicit value based coping. The vmPFC, medially placed in the frontal region of the prefrontal cortex, is reciprocally connected to sensory cortices and limbic structures and is an important center for motivation and emotional regulation, especially for moral emotions (Anderson et al., 2006; Berridge and Kringelbach, 2008; Damasio, 1994; et al., 1996; Price 1999). The vmPFC regulates moral decisions by allowing more subtle motivational and emotional factors to be weighted in judgments (Adolphs, 2009; Damasio, 1994; Greene, 2001; Greene and Haidt 2002; Mitchell, Banaji, and Macrae 2005; Moll et al., 2001). Adults with vmPFC damage fail to show autonomic responses to socially meaningful stimuli (Damasio, 1990), and fail to avoid disadvantageous choices (Bechara, Tranel, and Damasio 2000). Moreover, vmPFC is involved in inter-group processes such that it is more active in response to viewing members of in-groups (such as racial or social class in-groups) compared to stigmatized others (Harris and Fiske, 2006; Firat et al., 2017).

The dlPFC is the center for executive, top down functioning and goal directed behavior with connections to the temporal and parietal cortices to receive visual, somatosensory, and auditory information and the motor areas of the brain to coordinate movement (Firat and Hilton, 2000; Miller and Cohen, 2001; Miller and Cohen, 2019). For example, dlPFC plays a crucial role in working memory and multi-tasking (D’esposito et al., 1995; Cohen et al., 1997; Courtney et al., 1998). Patients who have damage to their dlPFC show inability to plan ahead and generate hypotheses, have trouble in flexibility tasks demanding flexibly shifting sets or changing tasks, have poor verbal fluency, and poor organizational and constructional strategies in learning new tasks (Milner 1963; Benton 1968; Jones-Gotman and Milner 1977; Stuss and Benson 1984; Waxman 2016). Moreover, some studies also suggest that the dlPFC has a role in a cognitive control mechanisms inhibiting racial bias (Stanley et al., 2008).

Based on this functional dissociation between the vmPFC and the dlPFC, I argue that we can identify the dissociable mechanisms for cultural coping strategies in response to ethno-racial stress in the human brain as 1) increased activation in the ventromedial prefrontal cortex (the brain region associated with moral intuitions and in-group identity, see Firat et al., 2017; 2019) when people adopt communal value based coping and 2) increased activation in the dorsolateral prefrontal cortex (brain region involved in top-down inhibition and executive control, e.g., Koechlin et al., 2003; MacDonald, 2000) when people employ agentic value based coping. I expect that both of these mechanisms will decrease psychophysiological stress responses (heart rate, heart rate variability, respiration and skin conductance) and thus potentially reduce allostatic load (McEwen and Stellar 1993).

**FIGURE 1:**
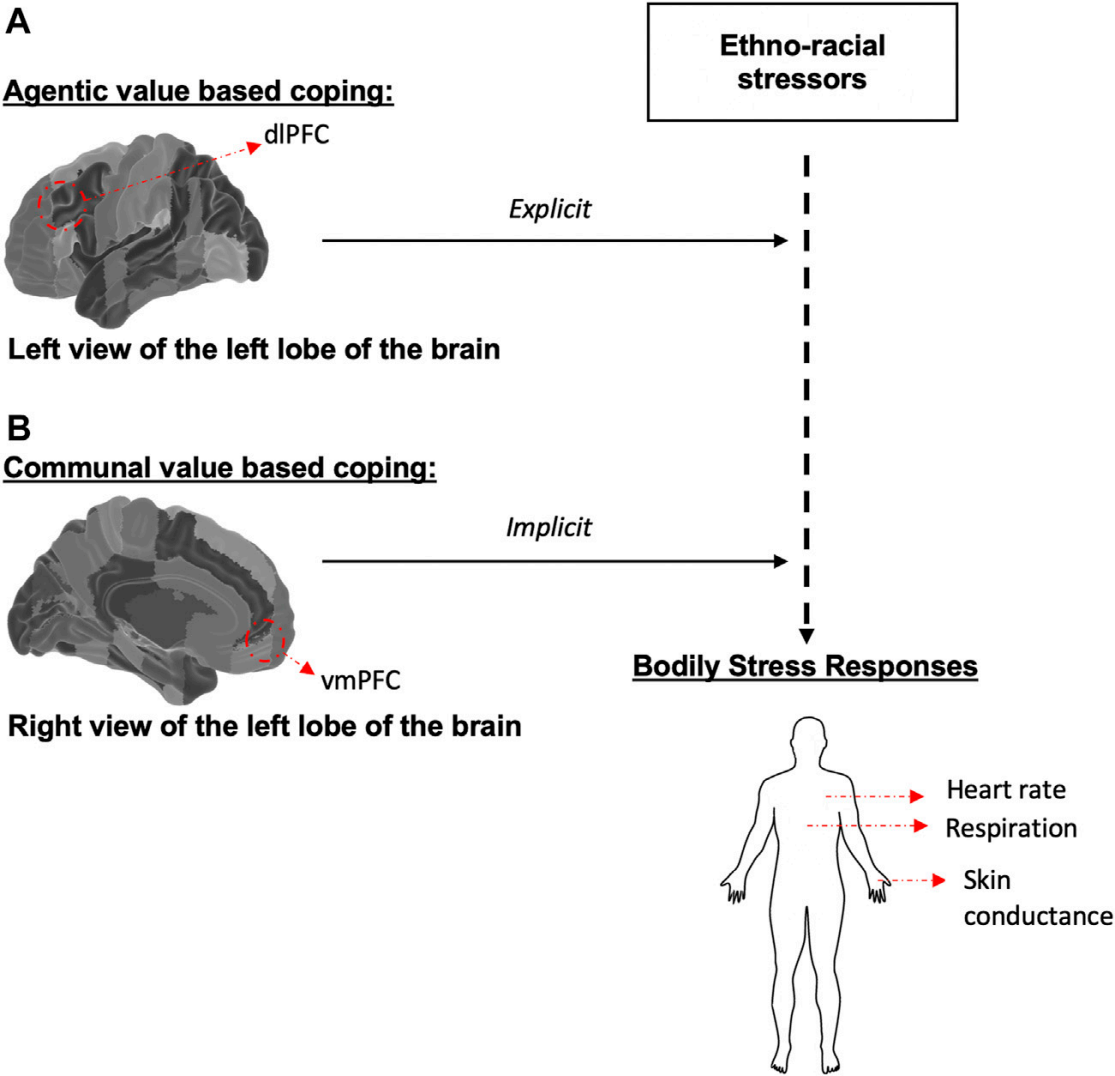
Illustration of the dual processes of value based coping. The brain atlases adapted from the Harvard-Oxford atlas developed at the Center for Morphometric Analysis (CMA), and distributed with the FMRIB Software Library (FSL) (Bakker et al., 2015), 3D Surface View (Majka et al., 2012).

In sum, as also illustrated in Figure 1, this paper proposes two value based coping styles: 1) an implicit motivational coping mechanism attenuating felt social pain via communal values, associated with ventromedial prefrontal cortex (vmPFC) activity and 2) another explicit coping mechanism providing more active and adaptive strategies to these difficult situations through agentic values, underlined by dorsolateral prefrontal cortex (dlPFC) activity. Again, this is not to say that individuals identifying with different groups will uniformly express the same coping styles and to the same extent; but rather, the argument I propose is that available patterns of practice shape the ways human brains respond to social or non-social environments, enabling people to co-create shared experiences (Roepstorff et al., 2010; Ma et al., 2014).

## An Empirical Illustration

In this section of the paper, I provide an empirical illustration of the dual process model of value coping mechanisms with some data from a functional Magnetic Resonance experiment that measured responses to social exclusion in seventeen Black and eleven White subjects while they were primed with agentic or communal values (for more details see Supplementary Table S1). Similar to the previous neurological research on social exclusion (e.g., Eisenberger et al., 2003; Kawomoto et al., 2012; Kumar et al., 2009; Masten et al., 2011; Sebastian et al., 2010), the experimental task measured neural activity inside an fMRI scanner while participants were playing a virtual ball tossing game (Cyberball) with two other players. All imaging data were obtained with a 3-tesla MRI scanner (Research Trio 3T).

Cyberball is an online or offline (pre-programmed) ball tossing game between two or more players represented with animated gifs (see attached Cyberball example pic) and has been widely used for research on ostracism, social exclusion, or rejection (Williams 2006; et al., 2012). As in previous Cyberball studies, participants were told prior to scanning that we were interested in “mental visualization” ability (to avoid the topic of social interaction), and that they would play a game of catch over the internet with two other players. They were instructed to imagine the experience as real as possible. In this game, social exclusion was simulated with inclusion or exclusion from the game with ball tosses. There were a total of three runs (agentic vs. communalistic vs. control). At the beginning of each run, the participants read a short paragraph describing three almost identical stories describing a trip to the city, beach or the countryside with equal number of pronouns and gave their answer on a screen with a finger key response. This cultural priming procedure, also referred to as the “pronoun circling task” has been shown to effectively prime agentic (individualistic) or communalistic (collectivistic) values in a number of previous behavioral as well as fMRI studies (Gardner et al., 1999; Harada et al., 2010; Oyserman, Coon, and Kemmelmeier, 2002; Oyserman and Lee, 2007). All priming conditions contained identical stories, except for the following target phrases: I, my, me for individualism; we, our, us for collectivism; and they, their, theirs as a control/comparison condition (see attached Supplementary Table S1 for details).

**FIGURE 2:**
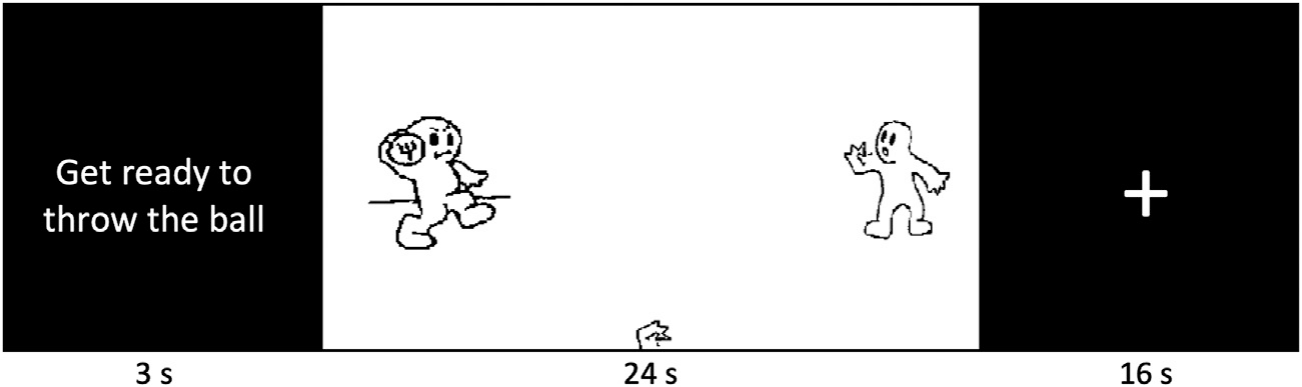
Cyberball paradigm.

After the value priming, there were a total of 12 blocks in each run (six each of inclusion and exclusion). In the inclusion condition, the participants were over-included (50% ball possession) in order for them to register systematic differences between the inclusion and exclusion conditions and for the exclusion condition they were partially or completely excluded (16% or 0% ball possession). Each block lasted 24 s, followed by a fixation cross (16 s) (see Figure 2). The order of inclusion and exclusion blocks were pseudorandomized with the constraint that the same block type was never presented more than twice in a row. Each block began with an instruction screen (3 s) telling participants to “Get ready to throw the ball”. Participants chose which of the other players to throw the ball to using a right index or middle finger key press response. Participants also marked each time one of the other players received the ball with a key press response in order to ensure that participants made motor responses during both inclusion and exclusion blocks and pay attention to the game. This cyberball paradigm is adapted from previous fMRI studies (see Sebastian et al., 2010; Kumar et al., 2009).

Data were first analyzed as individual level data and then combined into group level analyses on individual statistics. Data analysis presented here includes region of interest analyses (ROI) (for vmPFC and dlPFC brain regions) using AFNI (Analysis for Functional Neuroimaging) software. For each functional run, data were preprocessed to remove sources of noise and artifact. Data were corrected for differences in acquisition time between slices for each whole-brain volume, realigned within and across runs to correct for head movement, and co-registered with each participant's anatomical data. Normalized data were then spatially smoothed. Then, a deconvolution analysis was used to extract a hemodynamic response function for each subject. The effects of the conditions were modeled by box-car regressors convolved with the hemodynamic response function for 24 s for blocks of each trial type. Motion correction parameters were included as nuisance covariates and TRs with motion derivatives exceeding the Euclidian norm of 0.4 mm were censored in deconvolution analysis. Group-level analyses on the hemodynamic response estimates from individual statistics were conducted with a three way (race x inclusion/exclusion x values) Multi-Variate Modeling Approach (AFNI’s 3DMVM command) that applied ROI masks for dlPFC and vmPFC.

**FIGURE 3:**
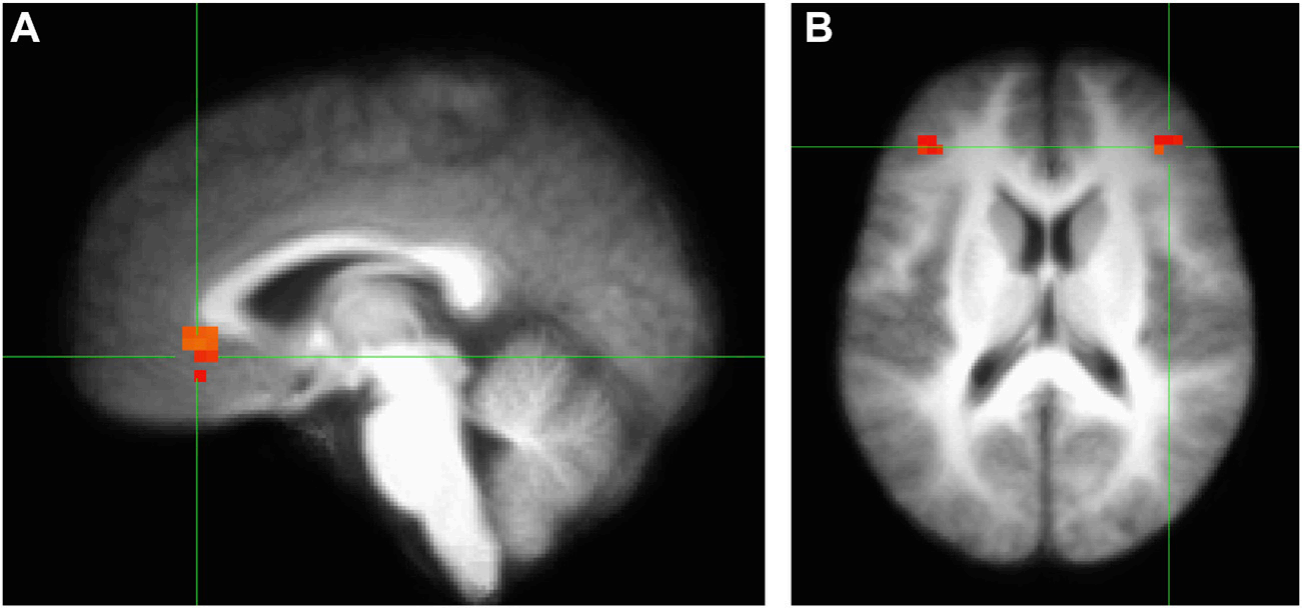
Increased activation of the vmPFC **(A)** and the dlPFC in conditions of interest. vmPFC cluster size = 21 MNI coordinates = 2, −32, −3 dlPFC left cluster size = 13 MNI coordinates = 36, −37, 19. dlPFC right cluster size = 9 MNI coordinates = −39, −30, 29 Uncorrected *p* = 0.01, corrected *p* = 0.05.

In Figure 3 present results from the contrasts of some main conditions of interest as they relate to the key arguments proposed by the theory outlined here: 1) communalistic values will recruit an implicit neural system including vmPFC activation and 2) agentic values will recruit an explicit neural system including dlPFC activation. I would like to emphasize that the results shown on Figure 3 are correlations (as most fMRI data are), focuses on ROI analysis (rather than whole brain) and do not include various main effects or different contrasts. In other words, these results are presented to demonstrate that a neurosociological approach is a viable one to study racial differences in values and coping and not as full-fledged results to draw empirical generalizations from. However, nonetheless these results depict a very interesting picture that is for the most part supportive of the theory proposed here. As brain activity is usually observed in relation to various tasks, I present here the contrasts between specific conditions to be able to isolate the activation in response to the conditions of interest that are not shared by other conditions. Accordingly, as can be seen on Figure 3, Panel **A**, there is greater vmPFC activation during communalism (vs other) and exclusion (vs inclusion) contrasts and greater dlPFC activation in agentic (vs communalistic) and exclusion (vs inclusion) contrasts in Black (vs White) respondents. This empirical illustration provides preliminary evidence for two potential mechanisms, one implicit and the other explicit, for the ways values might help racial minorities cope with the effects of social exclusion. These contrasts do not necessarily indicate that White respondents in the sample did not show similar responses. It simply meant that for Black respondents the findings were stronger. This makes sense given the context and history of United States race relations where marginalization, oppression and systemic exclusion of Black folk are hardened into the social fabric, causing a stress and anxiety overload.

## Future Directions/Conclusion

This paper seeks to advance current understandings of cultural and cognitive sociology and the dual process models through uncovering the ways in which values can attenuate responses to social exclusion through influencing implicit and explicit neurological processes. Recent research on dual process models has rejuvenated the interest on methodologies that go beyond traditional ethnography or survey methods to capture implicit and automatic cognitive and emotive processes of culture (e.g., Miles et al., 2019; Vaisey 2009). Social behavior and attitudes are especially complex and challenging to measure directly in the context of inter-racial or ethnic relationships (Pager and Shepherd 2008). Survey or interview responses might not only accurately reflect individuals’ beliefs due to social desirability but also might technically not be able to capture subtle and fast bodily reactions to social cues. The neurosociological methodologies proposed in this paper have the unique capacity to measure both fast and automatic and slow and controlled, thus avoiding social desirability issues. With this study, we are getting one step closer to a proper understanding of how culture shapes individual behavior implicitly and explicitly, detailing how distal social forces shape human mind and behavior.

As structurally and culturally shaped dispositions (Longest et al., 2013), values are well-situated for operationalizing the concept of cultural coping by embedding individuals in groups, organizations and broader cultural settings (Firat et al., 2018; Firat and McPherson, 2010). Building on previous research demonstrating that values moderate the negative effects of discrimination on well-being (Firat, 2017), I proposed and provided preliminary evidence that value orientations operate both through implicit/automatic (communal values) and explicit/controlled (agentic) values. The brain region related to automatic and emotional evaluations and processing (i.e., the vmPFC) was activated in Blacks in response to exclusion in communalistic conditions, whereas activation in the brain region most commonly associated with executive control and top-down processing (i.e., dlPFC) was related to agentic conditions during exclusion. Understanding the role of cultural values in stress responses is important in identifying a key contextual factor shaping individual responses, a mechanism often ignored by both psychologists and sociologists because it requires a thorough integration of macro and micro processes. By focusing on this macro-micro link with a neurosociological approach, this project makes a novel contribution to the body of knowledge on the effects of ethno-racial contact on stress. The mechanisms proposed in this paper are scalable to larger groups, other cultures, and identities in an increasingly diverse global society.

Future research applying this theoretical paradigm will have the capacity to identify ways for reducing ethno-racial disparities of stress and health. If the theory proposed here is further supported and augmented by empirical work, additional studies can identify specific value based intervention strategies for coping with stress in members of ethno-racial minority groups. While shared experiences of (interpersonal and institutional) discrimination and systematic social exclusion situate members of ethno-racial minority groups into oppressed and marginalized positions in the social hierarchy, values might serve as cultural, collective resources that can help improve resilience in the face of stressors. In this framework, values act as “a key cognitive capacity that can act as a mental resource for communities of color” (Firat, 2020: 77). If research can demonstrate culturally relevant implicit and explicit value strategies (for example through framing or messages) effective in buffering ethno-racial stress, these strategies could be adapted for protecting and promoting the health of members of vulnerable communities.

In conclusion, this paper has important implications for identifying the biomarkers for coping styles in response to ethnicity related stress by focusing on a sociological mechanism that operates both at the individual and macro level: value orientations. Previous research has focused on personal backgrounds such as previous racial contact or cross-ethnic friendships or psychological attributions such as self-esteem; but, no research has explored the macro societal and cultural dimensions underlying coping strategies at the micro level. The logical next step in this research paradigm would be to connect these neurological mechanisms with physiological stress responses to demonstrate the culture-brain-stress link. Responses to social interactions including ethno-racial interactions are bodily and cognitive phenomena; they are experienced and enacted through our bodies and our brains. Traditional experimental techniques like behavioral responses or survey methods are indirect ways of measuring these cognitive and bodily processes. Coupling brain imaging data with physiological data would be a spearheading attempt in linking biological mechanisms of ethno-racial relations to sociological understandings. Hopefully, this paper was able to offer a neurosociological research framework for future research seeking to expand our understandings of stress mechanism and response to social exclusion as they relate to value based coping strategies.

## Data Availability

The raw data supporting the conclusions of this article will be made available by the authors, without undue reservation.
