# Supplementary material for: A Neurosociological Theory of Culturally and Structurally Situated Cognition and Ethno-Racial Stress
Source: Front Sociol. 2021 Jun 11;6:695042. doi: 10.3389/fsoc.2021.695042 (PMC8225953; doi:10.3389/fsoc.2021.695042)
Supplement: Supplementary file 1 [file Table1.DOCX]

Supplementary Material

# Methodological Details

I implemented a 2 x 2 x 3 (race x exclusion x cultural values) factorial design, where race (Caucasian vs. African American) was a between level factor and social inclusion vs. exclusion and cultural values (individualism vs. collectivism vs. control/no value) were within level factors. Similar to the previous neurological research on social exclusion (e.g., Eisenberger et al. 2003; Kawomoto et al. 2012; Kumar et al. 2009; Masten et al. 2011; Sebastian et al. 2010), the experimental task measured neural activity inside an fMRI scanner while participants are playing a virtual ball tossing game (cyberball) with two other players. These two other players were actually a computer program written in E-prime with output simulating other players' ball tosses; however this information will be provided only after the experimental task is complete (see attached debriefing statement). Cyberball is an online or offline (pre- programmed) ball tossing game between two or more players represented with animated gifs (see attached Cyberball example pic) and has been widely used for research on ostracism, social exclusion, or rejection (Williams 2006, et al. 2012). As in previous Cyberball studies, participants were told prior to scanning that we were interested in mental visualisation ability (to avoid the topic of social interaction), and that they would play a game of catch over the internet with two other players.

**Fmri Parameters:**

Functional runs:

Voxel size: 2.8×2.8×2.8 mm

Slices: 40

Orientation: Transversal

FoV read: 224 mm

Slice thickness: 2.80mm

TR: 2000ms

TE: 30.0ms

Flip angle: 77 deg

T1 Anatomical:

Voxel size: 1×1×1 mm

Slices: 176

Orientation: Saggital

FoV read: 256 mm

Slice thickness: 1mm

TR: 2250ms

TE: 3.98ms

Flip angle: 9 deg

**Value Primes:**

Read the following text, and count the pronouns (I, me, my).

Read the following text, and count the pronouns (We, our, ours).

Read the following text, and count the pronouns (They, their, theirs).

*{For each prime, “I, me, my” will be replaced with other appropriate primes from the following paragraphs.}*

**Prime A**

I go to the city often. Before I go I like to anticipate what I will see. My anticipation fills me as I see the skyscrapers come into view. I allow myself to explore every corner, never letting an attraction escape me. My voice fills the air and street. My feeling touches all the places I have seen. I see all the sights, I window shop, and everywhere I go I see my reflection looking back at me in the glass of a hundred windows. I walk, I run, I let my imagination fly throughout my exploration. At nightfall I linger, my time in the city almost over. When finally I must leave, I do so knowing that I will soon return. The city belongs to me.

**Prime B**

I go to the beach often. Before I go I like to anticipate what I will see. My anticipation fills me as I see the palm trees come into view. I allow myself to explore every corner, never letting an attraction escape me. My noise fills the sea air and saltwater. My feeling touches all the places I have seen. I watch the sea gulls, I build sand castles, and everywhere I go I see my reflection looking back at me in the calm of the beautiful ocean water. I swim, I sunbathe, I let my imagination fly throughout my exploration. At nightfall I linger, my time at the beach almost over. When finally I must leave, I do so knowing that I will soon return. The beach belongs to me.

**Prime C**

I go to the countryside often. Before I go I like to anticipate what I will see. My anticipation fills me as I see the country road comes into view. I allow myself to explore every corner, never letting an attraction escape me. My voice fills the fields and the woods. My feeling touches all the places I have seen. I watch the blue skies, I run in the grassland, and everywhere I go I see my reflection looking back at me through peaceful water meadows. I breathe, I smell, I let my imagination fly throughout my exploration. At nightfall I linger, my time at the countryside almost over. When finally I must leave, I do so knowing that I will soon return. The countryside belongs to me.
